# Virtual Reality Simulation in Interprofessional Pediatric Cardiology Education

**DOI:** 10.7759/cureus.81181

**Published:** 2025-03-25

**Authors:** Lisa M Ring, Juliana DeBitetto, Jenhao J Cheng, Gregory K Yurasek

**Affiliations:** 1 Advanced Practice Providers, Children's National Hospital, Washington, DC, USA; 2 Pediatrics, George Washington University School of Medicine and Health Sciences, Washington, DC, USA; 3 Quality and Patient Safety, Children’s National Hospital, Washington, DC, USA; 4 Cardiac Critical Care, Children's National Hospital, Washington, DC, USA

**Keywords:** advanced practice providers, pediatric, pediatric cardiology, simulation-based continuing education (sbce), virtual reality simulation

## Abstract

Introduction

In acute care settings, pediatric congenital heart patients require comprehensive team-based care. Physicians and advanced practice providers (APPs) co-manage these patients in the same manner. However, there may be few opportunities to train together. Virtual reality (VR) simulation in the pediatric cardiology setting offers a portable, immersive experience and can be a cost-saving alternative to traditional manikin-based simulation. We sought to study the effectiveness of VR as a tool for providing interprofessional education to physicians and APPs working with cardiology patients in both the cardiac intensive care unit (CICU) and the emergency department (ED).

Methods

Physicians and APP pairs who clinically manage pediatric cardiology patients in the emergency department, cardiology acute care, and CICUs were identified to participate in two of eight VR simulations developed by a stand-alone pediatric hospital. The APP specialty was used to determine the appropriate selection of simulations for each provider pair. Four scenarios were designed in a virtual CICU, and four were in a virtual ED. Each physician-APP pair met for one hour, during which a 10-minute orientation, a five- to 10-minute simulation, a 10-minute debrief, and a second simulation were conducted. Each simulation was based on actual clinical situations, including five priority objectives for patient management to be met in five minutes or less, and whether a physician or APP initiated each objective was recorded. Following the VR simulations, participants completed a debrief and exit questionnaire.

Results

Seventeen physician-APP pairs participated in 33 simulations. An average of 3.4 objectives were met across all scenarios. Of the objectives met, 41% were initiated by an APP. Thirty-one participants completed post-simulation surveys. All agreed or strongly agreed that the VR environment enhanced their simulation experience and believed VR-based simulation could be useful for education in various pediatric settings.

Conclusion

Virtual reality simulation offers an immersive educational experience for providers across different professions within a pediatric cardiology setting. Further endeavors include evaluating bedside nurses' use of VR and comparing the use of VR with manikin-based approaches.

## Introduction

Effective multidisciplinary communication, collaboration, and teamwork are essential to delivering safe, high-quality patient care in acute hospital settings [1-3]. Interprofessional healthcare teams working collaboratively can reduce duplication, improve care coordination, and enhance safety and quality of care [4-7]. As changes and new challenges in healthcare have occurred, the role of pediatric acute care Advanced Practice Nurses (APN) has evolved to provide comprehensive care within the interdisciplinary care team [8,9] with increasing levels of autonomy and management of more complex patients [10]. Communication is a core component of leadership skills, which simulation-based training can support. Moore (2023) described a novel Advanced Practice Provider (APP) conceptual model highlighting the interrelatedness of clinical care, education, and professional development [10]. Advanced practice providers and physicians collaboratively provide clinical management for cardiology and post-operative cardiothoracic surgery patients in our heart institute (HI) and for patients with heart disease presenting to the emergency department (ED). To respond effectively and appropriately to the decompensating pediatric cardiac patient, providers should have the skills to quickly assess the clinical situation, provide needed interventions, and escalate care.

Simulation is a known teaching methodology that promotes learning and education and can help educators assess competency for identified skills and procedures. It also provides controlled, safe, and reproducible educational opportunities for team management and for practicing high-risk, low-frequency events [11,12]. High-fidelity simulation provides training for high-risk activities in a safe and effective manner [13] and is an educational staple for training in our cardiac intensive care unit (CICU). However, the maintenance of high-fidelity manikins can be expensive, cost thousands of dollars, and can be cumbersome to set up and operate [14,3]. Additionally, there is the cost of room or space for equipment use and storage, including task trainers, urinary catheters, central line kits, etc. [15], and equipment maintenance. Virtual reality (VR) is an alternative, potentially cost-effective simulation methodology being used increasingly in medical training and education [14, 16, 17]. Advanced practice providers are progressively being used to address healthcare shortages and demands [18] with increased levels of autonomy in caring for complex patients [10]. Virtual reality simulation has the potential to help support APP collaborative education in acute care areas. In their pilot VR work, Ralston et al. (2021) found that physicians unfamiliar with VR could engage in VR-simulated scenarios common to the CICU environment with minimal orientation and that VR simulation could be used when practicing how to manage a patient with hemodynamic compromise [14].

Simulation is a key component of learning in the CICU. Although APPs, nurses, and physicians participate in simulation learning, HI staff and ED APP staff do not attend educational sessions together. Logistically, the HI and ED provider clinical schedules made it difficult to schedule optimal education session times for both groups to attend at the same time. Additionally, when APPs and physicians participate together in manikin-based simulation, it is common for the physician to take the lead, with the result being a missed opportunity for the APP. Therefore, following an effort at our pediatric hospital utilizing VR simulation for CICU education among cardiology fellows, we were interested in broadening these sessions to include interprofessional teams, including fellows and APPs. Our primary goal was to explore the concept of VR simulation as an educational tool to determine the feasibility and effectiveness of providing interprofessional cardiology education to fellows and APPs from different disciplines. We anticipated that both APPs and fellows would participate in VR medical decision-making and that the assessment of objectives met to support this prediction.

## Materials and methods

This project was undertaken as a quality improvement initiative at a free-standing 300+ bed pediatric quaternary care, Magnet®-designated hospital from February to April of 2023 and did not constitute human subjects research. As such, it was not under the oversight of the institutional review board. De-identified data were kept on a password-locked computer and did not require advisory board oversight.

An empty 12 × 12-foot space was used to run each simulation. This was required to promote a safe space for the participants. We chose to utilize our patient monitoring space in the CICU. This area was a non-clinical ample open space to move around. It was also near the clinical space, allowing for a location within the HI. Prior to starting a VR scenario, two to three of the VR facilitators met for 30 minutes to set up the VR space and did a “run-through” to ensure there was consistency in documentation and provide debrief feedback. A 12 × 12-foot grid was defined using painters’ tape. The VR laptop, haptics, and Oculus® goggles (Meta, Menlo Park, CA) were evaluated and set up for use within this space. During the scenario, one facilitator ran the laptop, one facilitator ensured that the participants did not collide, and a third facilitator documented the objectives met during the scenarios and ran each scenario debrief.

All APPs working in the different HI specialty areas (clinic, acute care units, CICU) and the ED used SignUpGenius® (SignUpGenius, Inc., Charlotte, NC) to select a VR session to attend. All pediatric cardiology and pediatric critical care medicine (PCCM) fellows have to rotate through the CICU for at least four weeks. Seventeen fellows were selected to join the education session based on when their CICU rotation occurred, as this allowed for ease of scheduling and participation. An informational email about the VR sessions and SignUpGenius® was sent to both the ED APP (13 APPs) and HI APP blast groups (20 APPs). The APPs self-identified their availability to participate, and the physicians were a convenience group based on their clinical schedules. We selected 12 pm to 2 pm on Wednesdays, as this was a preferred time for both fellows and APPs. Cardiac intensive care unit-based APNs were assigned to the CICU-focused cases, and non-critical care APPs (APNs and physician assistants (PAs) from ED and CV surgery were assigned to the non-CICU cases. All participants received a 15-minute orientation to the VR equipment, haptics, and how the VR simulation sessions worked. First, we welcomed participants to the space and had them sign in and complete a demographic questionnaire including years of experience and provider profession. We then fitted their goggles and haptic devices and had each participant listen to the patient and feel the virtual pulses. Next, we oriented each participant to the technology and devices in the virtual room by showing them how to use the defibrillator, how to give medications, and how to provide bag-mask ventilation and oxygen therapy. Each participant was given a few minutes to practice before starting the actual scenario. All participants signed in and completed a post-scenario evaluation.

The two main facilitators included a CICU attending, who is the director of education and critical care simulation and also a certified healthcare simulation educator, and an APP education lead who facilitates the APP centralized onboarding simulation and has > 10 years of experience with pediatric simulation and cardiovascular postoperative management. The CICU attending and APP education lead facilitated all simulations.

Physicians and APP pairs were identified to participate in two of eight VR simulations developed by the hospital based on their APP specialty. We ensured that the scenarios we chose involved key concepts in pediatric cardiology and cardiac critical care and thus represented elements of core knowledge in these domains. Fellows’ specialties included pediatric cardiology, pediatric critical care, and pediatric cardiac critical care. APP fields included cardiology, CICU, cardiovascular (CV) surgery, and emergency medicine. All APPs working in the acute cardiology unit, CICU, and the ED were invited to participate as they are most likely to encounter pediatric cardiology patients needing acute management.

Of the eight different scenarios, four were designed in a virtual CICU and four were designed in a virtual ED. Scenarios in the CICU setting included low cardiac output syndrome (LCOS), pulmonary hypertensive crisis, post-operative hemorrhagic shock after a Norwood procedure, and unstable supraventricular tachycardia (SVT). The other four cases were set in a virtual ED and included a hypercyanotic spell in a patient with unrepaired tetralogy of Fallot, Blalock-Taussig-Thomas (BTT) shunt failure, neonatal coarctation of the aorta, and a presentation of myocarditis.

Simulations occurred on a weekly basis with each physician-APP pair having one hour to complete the orientation, two simulations, and a debrief. Lead roles were not preassigned for the scenario. Each five-minute simulation included five objectives to be met within five minutes or less, and the simulation team recorded whether a physician or APP initiated each objective (Table 1).

**Table 1:** Five objectives for each patient simulation under eight scenarios LCOS: low cardiac output state; JET: junctional ectopic tachycardia; PH: pulmonary hypertension; TOF: tetralogy of Fallot; SVT: supraventricular tachycardia; CXR: chest X-ray; Dx: diagnosis; CICU: cardiac intensive care unit; CV: cardiovascular; BP: blood pressure; BMV: bag valve mask

Scenario	Objective 1	Objective 2	Objective 3	Objective 4	Objective 5
LCOS JET	Staff assist called	Patient cooled	Sedation and/ or paralysis ordered	Atrial ECG ordered	Patient overdrive paced appropriately
Unstable SVT	Staff assist activated	Epinephrine dose asked to be drawn	Volume given	Defibrillator synched	Patient cardioverted
Postoperative Norwood	Staff assist activated	Epinephrine rate increased	Volume given	Calcium or blood given	Another vasopressor (dopamine, norepinephrine, or vasopressin) called for
PH crisis	Staff assist activated	Patient given BMV at 100% FiO2	Sedation and/or paralysis ordered	Nitric oxide ordered	Epinephrine given
TOF spell	Oxygen administered	Knees-to-chest attempted (by provider or nurse)	Volume given	Sodium Bicarbonate given	Phenylephrine given
Coarctation	BP measured in both upper/lower extremity	Coarctation Dx made	PGE started	Intubation discussed	CICU or CV surgery notified
Shunt occlusion	Oxygen given	Heparin bolus given	Volume given	Vasoactive infusion started	CICU or CV surgery notified
Myocarditis	Blood gas and a CXR ordered	Echo ordered	Myocarditis diagnosis made	Vasoactive started	CICU called for admission

Participants in each group were aware that their actions were being recorded. A 10-minute structured debrief was conducted after each simulation using the Plus-Delta technique [19], which included reviewing the objectives and discussing how the team interacted with the VR environment. Following the first simulation, a second simulation and debrief were conducted. After completing the VR simulations, participants completed an exit questionnaire, which included six responses on a Likert scale (Table 2) and testimonial responses.

**Table 2:** Post-simulation questionnaire * Negatively worded item

	Strongly disagree	Disagree	Neutral	Agree	Strongly agree
I had a lot of experience with virtual reality software before this session.	1	2	3	4	5
The virtual reality environment felt realistic.	1	2	3	4	5
The virtual reality environment enhanced my simulation experience.	1	2	3	4	5
The virtual reality medium distracted me from my medical decision-making.*	1	2	3	4	5
Virtual reality-based simulation can be useful for education in various pediatric settings.	1	2	3	4	5
I enjoyed this experience.	1	2	3	4	5

Statistical analysis

All programming and coding were performed by the medical VR simulation development company SimX (San Francisco, CA) with an iterative revision process in cooperation with the case authors over six months. Simulations were run on the SimX platform using Oculus Quest head-mounted displays (Meta) [14].

Project documents and data (objectives met) were reviewed and analyzed quantitatively by the project team. Simulation results and post-simulation survey data were analyzed and summarized using standard descriptive statistics, including frequencies, averages, sums, and percentages. We also used t-tests to compare average objectives initiated by the medical doctor (MD) and APP and chi-squared tests to compare initiated categories between CICU and ED. Statistical tests were two-sided, with the significance threshold set at p < 0.05. We did not apply missing imputation as the data were complete. All the analyses were performed using R Statistical Software version 4.1.2 for Windows (R Core Team 2023, R Foundation for Statistical Computing, Vienna, Austria). In addition to base R, we also used dplyr and ggplot2 packages for descriptive analysis (summaries) and data visualization (explorations).

## Results

A total of 17 physician-APP pairs participated in 33 simulations. For the CICU (Table 3), the overall average met is 3.5, and the APP’s average of 1.94 is slightly higher than MD’s average of 1.56 but was not significant (p = 0.406). On the other hand, for the ED (Table 4), the overall average met is 3.27, and the MD’s average of 2.53 is significantly higher than the APP’s 0.73 (p < 0.01). Combined, the CICU and ED overall average met is 3.4.

**Table 3:** Summary of objectives met across CICU simulations, and whether they were initiated by MD or APP Each simulation has five objectives to be initialized by MD or APP. MD: medical doctor (physician); APP: advanced practice provider; LCOS: low cardiac output state; PH: pulmonary hypertension' CICU: cardiac intensive care unit

Scenario	# Simulations	Average years of experience	Average objectives met (Max = 5)	Average objectives met initiated by MD	MD vs. APP	Average objectives met initiated by APP	
LCOS	4	11.88	4	2	=		2
Myocarditis	6	6.5	2.5	2	>		0.5
PH	4	10.75	4.5	1.5	<		3
Norwood	4	10.75	3.5	0.5	<		3
Total	18	9.58	3.5	1.56	<		1.94

**Table 4:** Summary of objectives met across ER simulations, and whether they were initiated by MD or APP Each simulation has five objectives to be initialized by MD or APP. MD: medical doctor (physician); APP: advanced practice provider; TOF: tetralogy of Fallot; SVT: supraventricular tachycardia; ER: emergency room

Scenario	# Simulations	Average years of experience	Average objectives met (Max = 5)	Average objectives met initiated by the MD	MD vs. APP	Average objectives met initiated by the APP	
TOF spell	7	6.29	3.29	2.86	>		0.43
Unstable SVT	4	11.25	3.25	2.25	>		1
Shunt occlusion	2	10	4.5	3.5	>		1
Coarctation	2	10	2	1	=		1
Total	15	8.6	3.27	2.53	>		0.73

For the overall study, 40% (66/165) of total objectives were initiated by MD and 27.9% of the objectives were initiated by an APP (46/165), where the difference is significant (p = 0.020). Medical doctors completed a strongly and significantly higher percentage of objectives than the APPs when participating in the ED simulations: 50.7% (38/75) vs. 14.7% (11/75), p < 0.001. However, MDs completed a slightly and insignificantly lower percentage than APPs when participating in the CICU simulations: 31.3% (28/90) vs. 38.9% (35/90), p = 0.274 (Table 5). Pairs collectively performed about the same when participating in the CICU or the ED scenarios with 3.5 vs. 3.3 average objectives met, respectively, p = 0.512 (Tables 3, 4). Physicians met more objectives than APPs in the myocarditis, hypercyanotic spell, unstable SVT, and shunt occlusion scenarios, whereas APPs met more objectives in the PH crisis and Norwood procedure scenarios. The two groups met the same number of objectives for the LCOS and coarctation scenarios (Tables 3-5). There were four average objectives met for the LCOS scenario for which the initiating person was not recorded (Table 3).

**Table 5:** Summary of total objectives by the initiated category MD: medical doctor (physician); APP: advanced practice provider; LCOS: low cardiac output state; PH: pulmonary hypertension; TOF: tetralogy of Fallot; SVT: supraventricular tachycardia; CICU: cardiac intensive care unit; ER: emergency room. The sum of MD and APP equals the total objectives met and % means row percentage.

Scenario	Initiated category, N (%)				Total simulated objectives (# simulations x 5 objectives)
	MD	APP	Neither	Unidentified	
LCOS	8 (40.0%)	8 (40.0%)	4 (20.0%)	0 (0.0%)	20
Myocarditis	12 (40%)	3 (10%)	11 (36.7%)	4 (13.3%)	30
PH	6 (30.0%)	12 (60.0%)	2 (10.0%)	0 (0.0%)	20
Norwood	2 (10.0%)	12 (60.0%)	6 (30.0%)	0 (0.0%)	20
TOF spell	20 (57.1%)	3 (8.6%)	12 (34.3%)	0 (0.0%)	35
Unstable SVT	9 (45.0%)	4 (20.0%)	7 (35.0%)	0 (0.0%)	20
Shunt occlusion	7 (70.0%)	2 (20.0%)	1 (10.0%)	0 (0.0%)	10
Coarctation	2 (20.0%)	2 (20.0%)	6 (60.0%)	0 (0.0%)	10
CICU total	28 (31.3%)	35 (38.9%)	23 (25.6%)	4 (4.4%)	90
ER total	38 (50.7%)	11 (14.7%)	26 (34.7%)	0 (0.0%)	75
Overall total	66 (40.0%)	46 (27.9%)	49 (29.7%)	4 (2.4%)	165

Cardiac intensive care unit APPs met more objectives on average than ED APPs for their respective scenarios. Cardiac intensive care unit APPs also met slightly more objectives than fellows (Table 3). Eighty-one percent of responders had no significant experience with VR before participating in these simulations (Table 6), and participants had an average of nine years of clinical experience (survey chart). More than 93% of participants reported the scenarios were realistic. A total of 31 participants completed post-simulation surveys, measuring the perception and feasibility of VR as a modality for pediatric clinical education. All participants agreed or strongly agreed that the VR environment enhanced their simulation experience and believed VR-based simulation could be useful for education in various pediatric settings.

**Table 6:** Post-simulation survey responses based on a Likert scale of one to five (strongly disagree to strongly agree) There are 31 returned surveys. Mean score = average of numerical responses (one to five for strongly disagree to strongly agree); Agreement % = percentage of agree and strongly agree responses (easier interpretation).

Survey question
I had a lot of experience with virtual reality software before this session.
The virtual reality environment felt realistic.
The virtual reality environment enhanced my simulation experience.
The virtual reality medium distracted me from my medical decision-making.
Virtual reality-based simulation can be useful for education in various pediatric settings.
I enjoyed this experience.

Comments from the participants included, “I really love the interdisciplinary aspect of the simulation,” “very interesting and beneficial simulation,” “incredibly helpful,” and “realistic. Although one learner noted it would be helpful for both providers to view labs and chest X-ray results, and another commented that at times the “visuals were difficult to manage for imaging labs,” participants generally responded “disagree” or “strongly disagree” to the response if VR distracted from medical decision-making. Another commented, “It makes simulation more fun and interactive by not seeing other people’s faces in real-time.” Additional comments also highlighted the absence of eye contact and body language.

## Discussion

To our knowledge, this is the first use of VR simulation for interprofessional cardiology learning in the pediatric environment. The sessions were interprofessional with both APPs and physicians identifying the clinical problem and choosing an intervention. We chose APPs from the HI and ED to participate in this project as these specialty groups manage pediatric cardiology emergencies. Although actual interprofessional teams include respiratory therapists and bedside nurses, we chose to first evaluate this educational methodology with our APPs and fellows as these providers generally have more flexibility to take part in scheduled offerings when on site. The project team included a CICU attending, an APN/educator, a nurse/medical student, and a simulation facilitator. The average years of participant experience were 9.1 years, indicating that although providers were not new to their roles, VR was a new learning experience for most. All participants agreed that VR simulation enhanced their simulation experience and that VR can be useful for education in pediatric settings.

The scenarios themselves were all based on actual cases and stemmed from a proactive organizational CICU cardiac arrest reduction quality improvement project in 2017 [20], and thus this effort aimed to provide a learning experience that targeted our patients’ needs. The successful meeting of the five objectives within the time frame provided an opportunity to help us determine the feasibility of the scenario and objectives. As direct caregivers, both physicians and APPs are likely to respond to the need for escalation of care for a cardiac patient in any environment, and thus working together in these scenarios provided a realistic experience. Pediatric cardiology emergencies need to be managed quickly, and a five-minute scenario provided enough time for a provider to emerge as a leader and for the moderators to evaluate whether the identified triage decisions were made in a timely manner. We found that APPs and fellows both contributed to clinical management decision-making. We did not have expectations of who would lead more of the objectives in the scenarios. We found that the CICU APPs emerged for leading the PH crisis and post-operative Norwood patients. Cardiac intensive care unit APPs only work in the CICU and are generally very familiar with PH crises and post-operative Norwood patients, and this may have impacted their ability to lead in these scenarios. The physicians work in many of the HI specialty areas and provide expert consultation for cardiac emergencies in the ED. Therefore, this group has more experience with myocarditis, hypercyanotic spells, unstable SVT, and shunt occlusion scenarios, which may explain why they emerged as leaders for those scenarios.

Those not working in the CICU or acute care unit generally have less experience with cardiac emergencies, and this might explain why these APPs met fewer objectives on average than their CICU and acute care counterparts. The absence of nonverbal communication inherent to the VR environment could potentially negatively impact performance, and this was noted by one participant. The impact of virtual and live eye contact is reduced when using VR [21]. Virtual reality can replicate non-verbal communication; however, it may not fully capture the realism of nonverbal cues [22, 23]. Our project did not evaluate if there was any impact on decision-making or teamwork. However, teams often rely on nonverbal cues, and the lack of live nonverbal cues could potentially have an effect or limit communication.

Although formal training and education for physicians and APPs follow different models, our results show the strength and potential effectiveness of their collaboration when managing acute and decompensating patients with congenital heart disease. Although we did not ask about future applications to these types of clinical situations, we know that multidisciplinary simulation training has been shown to promote effective behaviors such as a shared mental model, mutual performance monitoring, collaboration, and adaptability [24, 25]. In short, as high-quality interprofessional collaboration is essential during any actual patient crisis, it is essential that we also practice patient management together.

As we develop additional VR simulation modules, we can incorporate content specific to APP patient populations and integrate these modules into the onboarding process for our HI APPs. The standardized scenarios we present here could also be shared with providers in other pediatric heart centers and organizations that encounter pediatric cardiology emergencies in EDs, and they may be of particular use to providers who only see children with heart disease on occasion.

Over the past few months, we have expanded VR simulation education using the same objectives for our CICU bedside nurses, and each week we have three nurses and one rotating cardiology or PCCM fellow participate in these and other CICU-specific scenarios. We are continuing to study the effectiveness of this novel technology as we look to expand this effort to members of other professions also represented in the CICU, such as respiratory therapists, pharmacists, and child life specialists. The objective is to bring this novel type of learning to bedside staff with a continued focus on team dynamics and communication in addition to medical management.

In our organization, we are continuing to hire APPs in the acute care collaborative team setting, and providing a thorough onboarding process and opportunities for continuing education are fundamental to recruitment and retention. Using simulation, specifically VR scenarios, we can develop content that is applicable to these processes.

Limitations

Scheduling non-CICU APPs for these sessions provided a challenge, as lining up their clinical schedules with educational time was sometimes difficult. Also, as CICU APPs and fellows were always working clinically when they signed up for these sessions, we had to arrange for patient coverage during these sessions. In addition, the first few VR sessions required working out technological issues, which added to the session time and included wireless connectivity problems and issues with the headsets or haptics. Most of these issues were resolved with practice, and participants at most experienced only intermittent interruptions. They did not appear to be specific to the equipment, and often rebooting the technology fixed any issues. Once the issues were sorted, participants were generally able to run through the scenarios with little difficulty, although one learner commented that the “blinking floor” during the session was a little distracting. No session had to be canceled altogether for scheduling or technological issues. Participants were recruited by a pediatric cardiologist and former pediatric cardiovascular APN who is the organizational APP education lead. Although each scenario began with disclosures and a pre-brief clearly stating that performance on the scenarios would be confidential and would not have any effect on any clinical performance evaluation, the MD and APN who conducted the scenarios both have leadership roles in the hospital and thus, participant evaluations may have been influenced by this. To mitigate this bias, all survey data were anonymous. We recognize this project utilized a small sample size of convenience and that the content of the scenarios represents a small subset of the type of cardiac emergencies one might encounter in a pediatric ED or CICU, and both items could limit generalizability. Eye contact and body language are removed from the experience, and this could have an impact on communication. Last, as a post-experience skill assessment was not completed, no data are available regarding the participants’ retention of knowledge or skills as a result of taking part in this educational activity. However, future studies of this nature could address this.

## Conclusions

Virtual reality simulation supports experiential learning (repeated practice, direct observation, and feedback) in an engaging, team-oriented, safe learning environment, thus offering an opportunity for enhanced interprofessional education. It offers an immersive educational experience for providers across different professions within a pediatric cardiology setting. In our project, we found that APP and fellow participants each met a significant percentage of the objectives and appeared to value the experience. Future efforts will aim to expand this novel educational experience to additional providers, such as bedside nurses and respiratory therapists working in the growing field of pediatric cardiology.

## References

[1] Beebe P, Bawel-Brinkley K, O'Leary-Kelley C (2012). Observed and self-perceived teamwork in a rapid response team. J Nurses Staff Dev.

[2] Eddy K, Jordan Z, Stephenson M (2016). Health professionals' experience of teamwork education in acute hospital settings: a systematic review of qualitative literature. JBI Database System Rev Implement Rep.

[3] Lindamood KE, Weinstock P (2011). Application of high-fidelity simulation training to the neonatal resuscitation and pediatric advanced life support programs. Newborn Infant Nurs Rev.

[4] Hurlock-Chorostecki C, Forchuk C, Orchard C, Reeves S, van Soeren M (2013). The value of the hospital-based nurse practitioner role: development of a team perspective framework. J Interprof Care.

[5] McLaney E, Morassaei S, Hughes L, Davies R, Campbell M, Di Prospero L (2022). A framework for interprofessional team collaboration in a hospital setting: advancing team competencies and behaviours. Healthc Manage Forum.

[6] Reeves S, Lewin S, Espin S, Zwarenstein M (2010). Interprofessional Teamwork for Health and Social Care.

[7] Reeves S, Pelone F, Harrison R, Goldman J, Zwarenstein M (2017). Interprofessional collaboration to improve professional practice and healthcare outcomes. Cochrane Database Syst Rev.

[8] Jones MB, Tucker D (2016). Nursing considerations in pediatric cardiac critical care. Pediatr Crit Care Med.

[9] Ring LM, Cinotti J, Hom LA (2023). A quality improvement initiative to improve pediatric discharge medication safety and efficiency. Pediatr Qual Saf.

[10] Moore EF (2023). Development of an advanced practice conceptual model. J Nurse Practice.

[11] Kory PD, Eisen LA, Adachi M, Ribaudo VA, Rosenthal ME, Mayo PH (2007). Initial airway management skills of senior residents: simulation training compared with traditional training. Chest.

[12] Cheng A, Duff J, Grant E, Kissoon N, Grant VJ (2007). Simulation in paediatrics: an educational revolution. Paediatr Child Health.

[13] Cooper A (2015). High-fidelity simulation for neonatal nursing education: an integrative review of the literature. Neonatal Netw.

[14] Ralston BH, Willett RC, Namperumal S (2021). Use of virtual reality for pediatric cardiac critical care simulation. Cureus.

[15] Senvisky JM, McKenna RT, Okuda Y (2025). Financing And Funding A Simulation Center. https://www.ncbi.nlm.nih.gov/books/NBK568786/.

[16] Zhao G, Fan M, Yuan Y, Zhao F, Huang H (2021). The comparison of teaching efficiency between virtual reality and traditional education in medical education: a systematic review and meta-analysis. Ann Transl Med.

[17] Yurasek GK, Fortkiewicz J, Duelley C (2023). Interprofessional extracorporeal membrane oxygenation cardiopulmonary resuscitation simulations aimed at decreasing actual cannulation times: a longitudinal study. Simul Healthc.

[18] (2025). Quantifying the cost of advanced practice provider turnover. https://sullivancotter.com/wp-content/uploads/2020/02/Quantifying-the-Cost-of-Advanced-Practice-Provider-Turnover.pdf.

[19] Kolbe M, Grande B, Lehmann-Willenbrock N, Seelandt JC (2023). Helping healthcare teams to debrief effectively: associations of debriefers' actions and participants' reflections during team debriefings. BMJ Qual Saf.

[20] Riley CM, Diddle JW, Harlow A (2022). Shifting the paradigm: a quality improvement approach to proactive cardiac arrest reduction in the pediatric cardiac intensive care unit. Pediatr Qual Saf.

[21] AH Syrjämäki, P Isokoski, V Surakka (2020). Eye contact in virtual reality. A psychophysiological study. Comput Hum Behav.

[22] D Maloney, Freeman G, Wohn DY (2020). “Talking without a voice”: understanding non-verbal communication in social virtual reality. Proc ACM Hum Comput Interact.

[23] E Dzardanova, V Nikolakopoulou, V Kasapakis, Vosinakis S, Xenakis I, Gavalas D (2024). Exploring the impact of non-verbal cues on user experience in immersive virtual reality. Comput Anim Virtual Worlds.

[24] Ryan A, Rizwan R, Williams B, Benscoter A, Cooper DS, Iliopoulos I (2019). Simulation training improves resuscitation team leadership skills of nurse practitioners. J Pediatr Health Care.

[25] Clements A, Curtis K, Horvat L, Shaban RZ (2015). The effect of a nurse team leader on communication and leadership in major trauma resuscitations. Int Emerg Nurs.

